# Association between asthma and cardiovascular disease: evidence from the national health and nutrition examination survey 1999–2018

**DOI:** 10.3389/fcvm.2024.1367576

**Published:** 2024-12-20

**Authors:** Biao Peng, Wenjing Zhao, Fang Wan, Zhonghai Ji, Runkun Luo, Sheng Wang, Anhua Cao, Zhichao Yang, Da Liu, Changchun Tang, Ping Deng

**Affiliations:** ^1^ Department of Pulmonary and Critical Care Medicine, The Affiliated Changsha Central Hospital, Hengyang Medical School, University of South China, Changsha, Hunan, China; ^2^ Department of Cardiovascular Medicine, The Affiliated Changsha Central Hospital, Hengyang Medical School, University of South China, Changsha, Hunan, China; ^3^ Department of Neurology, The Affiliated Changsha Central Hospital, Hengyang Medical School, University of South China, Changsha, Hunan, China

**Keywords:** asthma, stroke, heart failure, angina pectoris, cardiovascular diseases, NHANES

## Abstract

**Background:**

Cardiovascular disease(CVD) remains a significant global challenge. Asthma, which is characterized by airway hyperresponsiveness and reversible and limited airflow, plays an important role in cardiovascular diseases. This study aimed to investigate the association between asthma and CVD.

**Methods:**

This cross-sectional study included demographic, laboratory, and questionnaire data from the National Health and Nutrition Examination Survey (NHANES) 1999–2018. CVD included stroke, congestive heart failure, coronary heart disease, and angina. Multiple logistic regression models were used to detect the association between asthma and the prevalence of CVD, adjusting for age, gender, race, education level, body mass index, ratio of family income to poverty, smoking exposure, drinking exposure, diabetes history, hypertension history, chronic obstructive pulmonary disease (COPD) history, and chronic kidney disease (CKD) history. A subgroup analysis was performed to investigate the association between asthma and CVD in different populations.

**Results:**

In total, 16,807 participants were included in this study, including 2,446 who reported having asthma. Compared with participants without asthma, the prevalence of stroke in those with asthma was increased by 1.607 times; the prevalence of congestive heart failure was increased by 1.911 times. Asthma significantly increased the prevalence of stroke among participants aged 18–44 years old, with a BMI 18.50–29.99 kg/m^2^, with low education levels, and with a PIR < 1.00. Asthma also increased the prevalence of angina in females, non-Hispanic Blacks, participants aged 45–59 years old, with a BMI ≥ 30.00 kg/m^2^, and with a PIR < 1.00. The prevalence of congestive heart failure was positively associated with asthma in non-Hispanic Whites or Blacks, participants aged ≥45 years old, with a BMI 25.00–29.99 kg/m^2^, with a PIR < 1.00, and with a low or middle education level.

**Conclusion:**

Asthma significantly increases the prevalence of stroke, congestive heart failure. Patients with asthma should be monitored for CVD, including stroke and congestive heart failure.

## 1 Introduction

Cardiovascular disease (CVD) is a leading cause of global mortality and a significant factor affecting patients’ quality of life. Although deaths from CVD are more prevalent in middle-income countries, recent data from the American Heart Association indicate that 928,741 individuals died from CVD in the United States in 2020 (1). CVD also impose a substantial economic burden on society. Between 2018 and 2019, the overall direct and indirect costs attributed to CVD reached $407.3 billion, accounting for 12% of the nation's total healthcare expenditure (2), and this figure continues to rise on a global scale (3, 4).

The treatment and management of CVD are focal points of global attention and pose significant challenges worldwide. The critical interactions between respiratory diseases and the cardiovascular system have been reported in several previous studies. Chronic obstructive pulmonary disease is associated with hypertension (5), myocardial infarction (6), atrial fibrillation (7), and heart failure (8). Recent studies reported a positive association between asthma and CVD. A Multiethnic cohort study demonstrated a significantly higher prevalence of CVD among patients with asthma than among those without asthma (9). A prospective population-based study conducted in Norway revealed a 38% higher risk of atrial fibrillation in individuals diagnosed with asthma (10). Additionally, findings from another study (11) indicated that patients with asthma exhibit an increased risk of cerebrovascular diseases [risk ratio (RR) 1.20, 95% CI (1.15–1.25)], coronary artery disease [RR 1.40, 95% CI (1.35–1.45)], and heart failure [RR 2.14, 95% CI (2.06–2.22)]. However, the results of two Mendelian randomized trials suggested that asthma does not significantly increase the prevalence of coronary artery disease (12, 13). Another large-scale longitudinal study in South Korea reported that asthma is a risk factor for stroke (14). Variations in race and socioeconomic status may contribute to the differences observed in the outcomes of these studies.

Although the Global Initiative for Asthma and World Heart Federation have provided optimal treatment and management strategies for patients with asthma and cardiovascular diseases worldwide, the annual prevalences of asthma and CVD continue to rise in most countries. Therefore, this population-based study using data from the NHANES was conducted to better understand the association between asthma and CVD.

## 2 Methods

### 2.1 Study population

All data were sourced from the National Health and Nutrition Examination Survey (https://wwwn.cdc.gov/nchs/nhanes/Default.aspx) conducted by the United States National Center for Health Statistics. This study aimed to evaluate the health and nutritional statuses of adults and children in the United States. Data from 1999 to 2018, prior to the Coronavirus Disease 2019 pandemic, were used to exclude any potential impacts of the novel coronavirus on the study outcomes.

**Figure 1:**
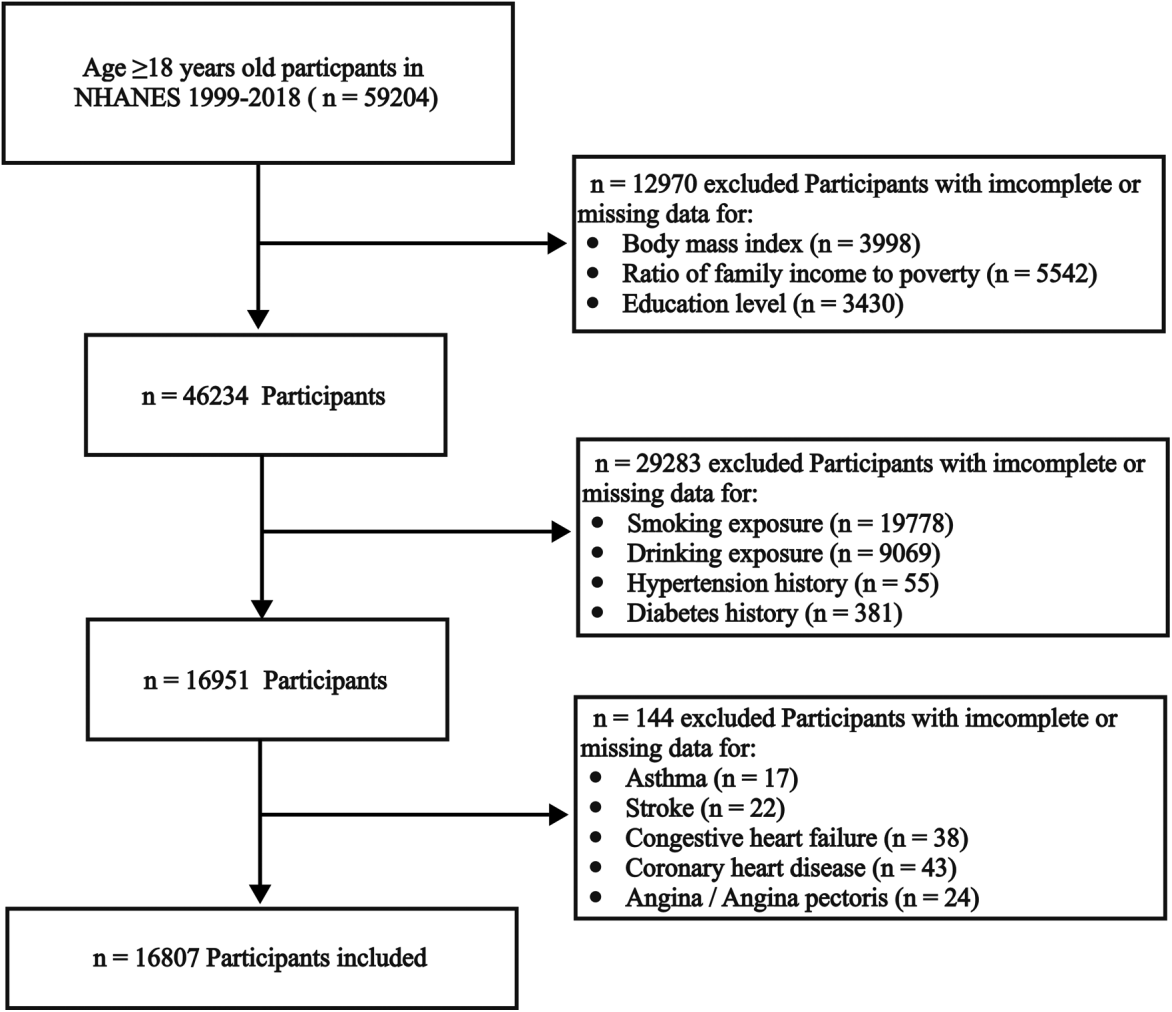
Flowchart of participant filtration in NHANES 1999–2018.

A total of 59,204 participants aged ≥18 years were included in the NHANES between 1999 and 2018. Participants with incomplete or missing data for body mass index (BMI), ratio of family income to poverty (PIR), or education level (*n* = 12,970); incomplete or missing data for smoking exposure, drinking exposure, hypertension history, or diabetes history (*n* = 29,283); or incomplete or missing data for the questionnaires “have you ever been told had stroke,” “have you ever been told had congestive heart failure,” “have you ever been told had coronary heart disease,” or “have you ever been told had angina/angina pectoris” (*n* = 144) were excluded from the study. Finally, 16,807 participants were included in the study (Figure 1).

### 2.2 Definition of asthma and cardiovascular events

Individuals who responded “Yes” to the questionnaire item, “Has a doctor or other health professional ever told you that you have asthma?” were considered to have asthma.

In this study, CVD included were stroke, congestive heart failure, coronary heart disease, and angina/angina pectoris. The participants were considered to have a history of CVD if they answered “Yes” to any of the following questions in the NHANES questionnaire: “Has a doctor or other health professional ever told you that you had congestive heart failure?”, “Has a doctor or other health professional ever told you that you had coronary heart disease?”, “Has a doctor or other health professional ever told you that you had angina, also called angina pectoris?”, and “Has a doctor or other health professional ever told you that you had a stroke?”.

### 2.3 Evaluation of covariates

According to preformed study (15, 16), this study selected 12 indicators as covariates. Demographic data included age, gender, race, educational level, and PIR. Examination and questionnaire data included BMI, smoking exposure, drinking exposure, hypertension history, diabetes history, chronic obstructive pulmonary disease (COPD) history, and chronic kidney disease (CKD) history.

(a) **Age:** presented as a continuous variable.
(b) **Gender:** categorized into male and female categories.
(c) **Race:** divided into four groups: Mexican American, non-Hispanic white, non-Hispanic black, and others.
(d) **Educational level:** divided into high school or below, some college, and college graduate or above.
(e) **PIR:** divided into three groups: below 1.00, between 1.00 and 1.99, and ≥2.00.
(f) **BMI:** according to the World Health Organization's 2000 BMI guidelines (17), participants were categorized as obese (≥30.00 kg/m^2^), overweight (25.00–29.99 kg/m^2^), normal weight (18.50–24.99 kg/m^2^), or underweight (<18.50 kg/m^2^).
(g) **Drinking exposure:** based on the annual alcohol consumption frequency (cut-off value, 12 times) or average monthly frequency (cut-off value, 1 time), participants were divided into those with drinking exposure and those without drinking exposure.
(h) **Smoking exposure:** based on the total amount of tobacco smoked (cut-off value, 100 cigarettes), participants were divided into smoking exposure and no smoking exposure.
(i) **Hypertension and diabetes history:** according to the responses to the questionnaire, participants were divided into groups with and without a history of hypertension and with or without a history of diabetes.
(j) **COPD history**: Participants were classified as having COPD if they answered “Yes” to any of the following questions: “Have you ever been told that you have emphysema?”, “Have you ever been told that you have chronic bronchitis?”, or “Have you ever been told that you have chronic obstructive pulmonary disease?” (18).
(k) **CKD history:** The estimated glomerular filtration rate (eGFR) values were calculated using the CKD-EPI formula for different genders, ages, and races (19). An eGFR < 60 ml/min/1.73 m^2^ was considered to indicate the presence of CKD.

### 2.4 Statistical analyses

All statistical analyses were conducted using IBM SPSS Statistics 26 and EmpowerStats. Survey weights, strata, and primary sampling units were used for all analyses to accommodate the complex and multi-unit survey design. Continuous variables are presented as mean and standard deviation (MD ± SD), while categorical variables are presented as percentages. The baseline characteristics of the participants with asthma were analyzed using paired *t*-tests for continuous variables and chi-squared tests for categorical variables. After adjusting for covariates, multivariate logistic regression was used to explore the association between asthma and CVD. Statistical significance was set at *P* < 0.05.

## 3 Results

### 3.1 Baseline characteristics of participants

Among the 16,807 participants included in this study, 2,446 (15.20%) had a self-reported history of asthma. The baseline characteristics of the study participants are presented in Table 1. Asthma was more prevalent among participants aged 18–44 years, females, non-Hispanic Black individuals, obese individuals, individuals with a PIR < 2.00, individuals with a college education, and those with a history of COPD, hypertension and diabetes. Compared with non-asthmatic participants, those with asthma had higher prevalences of stroke (3.53%), congestive heart failure (3.43%), and angina (3.02%).

**Table 1:** Characteristics of asthma and non-asthma participants.

Characteristics	Asthma (*n* = 2,446)	Non-asthma (*n* = 14,361)	*P* value
Age (%)			<**0**.**0001**
18-44	1,253 (54.94)	6,397 (48.04)	
45-59	612 (27.20)	3,640 (29.35)	
≥60	581 (17.87)	4,324 (22.62)	
Gender (%)			<**0**.**0001**
Male	1,172 (44.83)	8,423 (55.99)	
Female	1,274 (55.17)	5,938 (44.01)	
Race (%)			<**0**.**0001**
Mexican American	203 (4.14)	2,291 (7.35)	
Non-Hispanic White	1,283 (73.63)	7,169 (73.70)	
Non-Hispanic Black	554 (10.81)	2,666 (8.74)	
Other	406 (11.42)	2,235 (10.21)	
BMI (kg/m^2^)			<**0**.**0001**
18.50-24.99	43 (1.63)	229 (1.55)	
18.50-24.99	643 (28.76)	4,201 (30.41)	
25.00-29.99	732 (28.94)	4,994 (34.03)	
≥30.00	1,028 (40.67)	4,934 (34.00)	
PIR			<**0**.**0001**
<1.00	562 (16.62)	2,559 (11.85)	
1.00-1.99	670 (21.35)	3,593 (18.77)	
≥2.00	1,214 (62.03)	8,209 (69.38)	
Education level			**0**.**0062**
High school or below	1,072 (37.83)	6,806 (40.21)	
Some college	889 (38.15)	4,439 (32.70)	
College graduate or above	485 (25.02)	3,116 (25.06)	
Drinking exposure			0.6379
Yes	2,203 (90.87)	13,126 (91.25)	
No	243 (9.13)	1,235 (8.75)	
Smoking exposure			0.2642
Yes	2,105 (85.53)	12,231 (84.17)	
No	341 (14.47)	2,130 (15.83)	
Hypertension history			**0**.**0062**
Yes	900 (32.13)	4,594 (28.68)	
No	1,546 (67.87)	9,767 (71.32)	
Diabetes history			**0**.**0284**
Yes	310 (8.59)	1,372 (7.19)	
No	2,136 (91.41)	12,989 (92.81)	
COPD			<**0**.**0001**
Yes	595 (24.33)	768 (5.35)	
No	1,851 (75.67)	13,593 (94.65)	
CKD			**0**.**0468**
Yes	124 (5.34)	882 (6.42)	
No	2,197 (94.66)	12,848 (93.58)	
Have stroke (%)			<**0**.**0001**
Yes	117 (3.53)	384 (1.89)	
No	2,329 (96.48)	13,977 (98.11)	
Have congestive heart failure (%)			<**0**.**0001**
Yes	109 (3.43)	320 (1.47)	
No	2,337 (96.57)	14,041 (98.53)	
Have coronary heart disease (%)			0.9581
Yes	105 (3.35)	576 (3.33)	
No	2,341 (96.65)	13,785 (96.67)	
Have angina/angina pectoris (%)			**0**.**0010**
Yes	90 (3.02)	384 (1.89)	
No	2,356 (96.98)	14,038 (98.18)	

Bold values indicate statistical significance.

BMI, body mass index; PIR, the ratio of family income to poverty; COPD, chronic obstructive pulmonary disease; CKD, chronic kidney disease.

### 3.2 Associations of asthma and CVD

Table 2 presents the association between asthma and cardiovascular diseases (CVD). In the crude model, individuals with asthma had a higher prevalence of stroke [odds ratio (OR) 1.688, 95% confidence interval (CI) (1.359–2.098)], coronary heart disease [OR 1.965, 95% CI (1.536–2.5513)], and angina [OR 1.545, 95% CI (1.185–2.016)], but a lower prevalence of congestive heart failure [OR 0.699, 95% CI (0.544–0.897)].

**Table 2:** Associations between asthma and cardiovascular diseases.

Cardivascular disease	Crude model		Model Ⅰ		Model Ⅱ		Model Ⅲ	
	OR (95% CI)	*P* value	OR (95% CI)	*P* value	OR (95% CI)	*P* value	OR (95% CI)	*P* value
Stroke								
Yes	1.688 (1.359–2.098)	<**0**.**0001**	1.899 (1.520–2.372)	<**0**.**0001**	1.837 (1.469–2.298)	<**0**.**0001**	1.607 (1.213–2.129)	**0**.**0028**
No	Ref		Ref		Ref		Ref	
Congestive heart faliure								
Yes	0.699 (0.544–0.897)	**0**.**0049**	2.199 (1.708–2.832)	**0**.**0002**	2.082 (1.615–2.684)	<**0**.**0001**	1.911 (1.480–2.467)	**0**.**0010**
No	Ref		Ref		Ref		Ref	
Coronary heart disease								
Yes	1.965 (1.536–2.513)	<**0**.**0001**	0.894 (0.690–1.159)	0.3985	0.896 (0.691–1.161)	0.4053	0.861 (0.665–1.115)	0.2568
No	Ref		Ref		Ref		Ref	
Angina/Angina pectoris								
Yes	1.545 (1.185–2.016)	**0**.**0013**	1.676 (1.520–2.372)	<**0**.**0001**	1.609 (1.224–2.113)	**0**.**0006**	1.285 (0.953–1.733)	0.1006
No	Ref		Ref		Ref		Ref	

Bold values indicate statistical significance.

OR, odd ratio; 95% CI, 95% confidence interval; Ref, reference. Model I: Adjusted for age, gender and race. Model Ⅱ: Further adjusted for BMI and PIR. Model Ⅲ: Further adjusted for smoking exposure, drinking exposure, hypertension history, diabetes history, COPD history, and CKD history.

After adjusting for age, gender, and race, Model I showed an elevated prevalence of stroke [OR 1.899, 95% CI (1.520–2.372)], congestive heart failure [OR 2.199, 95% CI (1.708–2.832)], and angina [OR 1.676, 95% CI (1.520–2.372)]. Model II further adjusted for BMI and PIR, with the results remaining stable. Model III, which adjusted for all covariates, demonstrated that asthma was associated with an increased incidence of stroke [OR 1.607, 95% CI (1.213–2.129)] and congestive heart failure [OR 1.911, 95% CI (1.480–2.467)].

### 3.3 Subgroup analysis of the association between asthma and CVD

**Figure 2:**
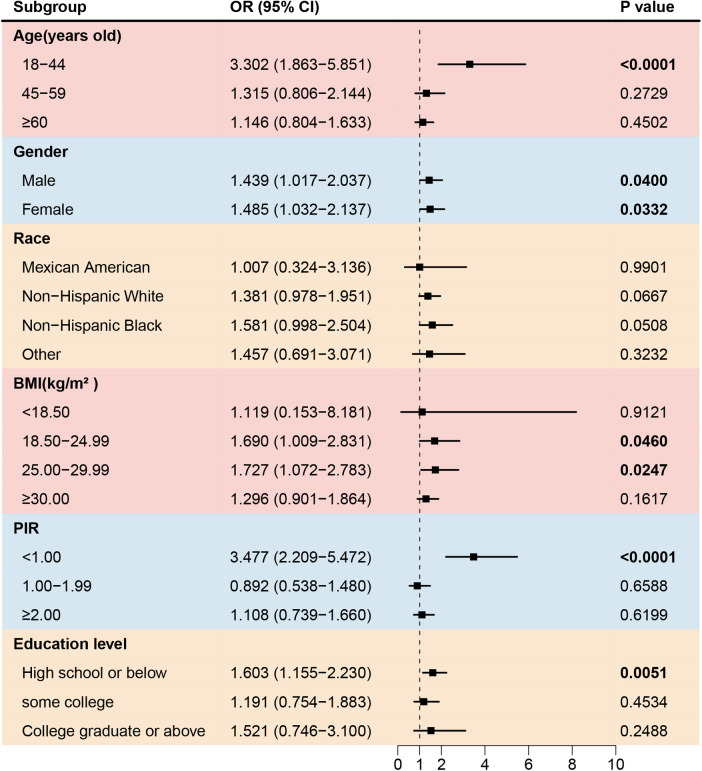
Forest plot of association between asthma and the prevalence of stroke. BMI, body mass index; PIR, the ratio of family income to poverty.

**Figure 3:**
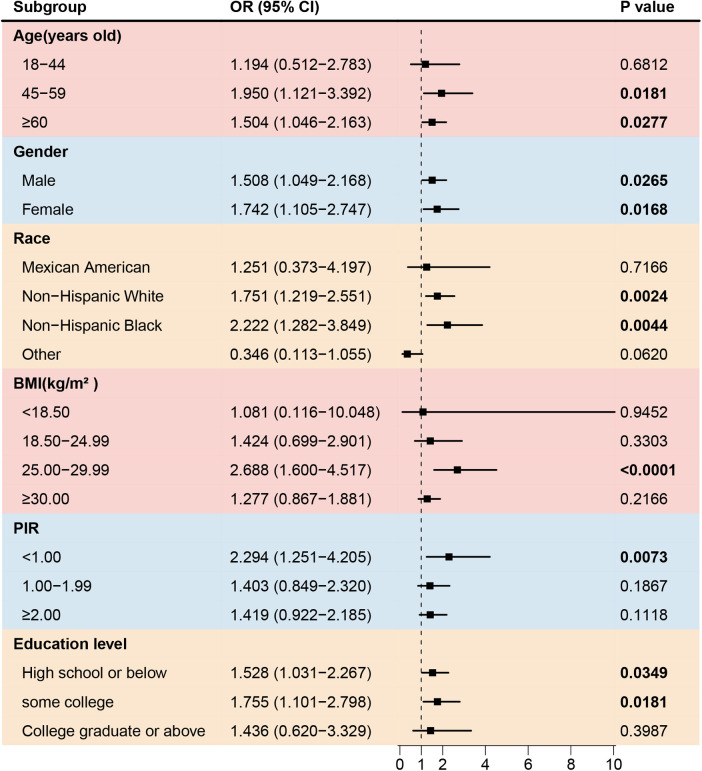
Forest plot of association between asthma and the prevalence of congestive heart failure. BMI, body mass index; PIR, the ratio of family income to poverty.

To further investigate the association between asthma and cardiovascular disease in different populations, subgroup analyses were conducted, as shown in Table 3. Asthma significantly increased the prevalence of stroke among participants aged 18–44 years old, with a BMI 18.50–29.99 kg/m^2^, with low education levels, and with a PIR < 1.00 (Figure 2). No gender-based associations were identified between asthma and stroke. The prevalence of congestive heart failure was positively associated with asthma among non-Hispanic Whites or Blacks, participants aged ≥45 years old, with a BMI 25.00–29.99 kg/m^2^, with a PIR < 1.00, and with a low or middle education level. (Figure 3). There were no significant differences between gender-based groups.

**Table 3:** Subgroup analysis of the association between asthma and cardiovascular disease.

	Stroke		Congestive heart failure		Coronary heart disease		Angina/Angina pectoris	
	OR (95% CI)	*P* value	OR (95% CI)	*P* value	OR (95% CI)	*P* value	OR (95% CI)	*P* value
Age(years old)								
18–44	3.302 (1.863–5.851)	<**0**.**0001**	1.194 (0.512–2.783)	0.6812	1.939 (0.699–2.086)	0.5206	2.179 (0.984–4.821)	0.0547
45–59	1.315 (0.806–2.144）	0.2729	1.950 (1.121–3.392)	**0**.**0181**	0.694 (0.380–1.268)	0.2352	2.223 (1.270–3.891)	**0**.**0052**
≥60	1.146 (0.804–1.633)	0.4502	1.504 (1.046–2.163)	**0**.**0277**	0.883 (0.631–1.235)	0.4681	0.879 (0.584–1.323)	0.5358
Gender								
Male	1.439 (1.017–2.037)	**0**.**0400**	1.508 (1.049–2.168)	**0**.**0265**	0.876 (0.624–1.229)	0.4430	1.032 (0.686–1.553)	0.8786
Female	1.485 (1.032–2.137)	**0**.**0332**	1.742 (1.105–2.747)	**0**.**0168**	0.715 (0.424–1.205)	0.2075	1.709 (1.090–2.680)	**0**.**0196**
Race								
Mexican American	1.007 (0.324–3.136)	0.9901	1.251 (0.373–4.197)	0.7166	0.593 (0.168–2.097)	0.4176	1.759 (0.567–5.456)	0.3279
Non-Hispanic White	1.381 (0.978–1.951)	0.0667	1.751 (1.219–2.5514)	**0**.**0024**	0.965 (0.653–1.424)	0.3380	1.381 (0.978–1.951)	0.8560
Non-Hispanic Black	1.581 (0.998–2.504)	0.0508	2.222 (1.282–3.849)	**0**.**0044**	0.738 (0.374–1.458)	0.3820	2.715 (1.378–5.348)	**0**.**0039**
Other	1.457 (0.691–3.071)	0.3232	0.346 (0.113–1.055)	0.0620	1.137 (0.478–2.702)	0.7713	1.826 (0.782–4.266)	0.1641
BMI(kg/m^2^)								
<18.50	1.119 (0.153–8.181)	0.9121	1.081 (0.116–10.048)	0.9452	1.684 (0.196–14.454)	0.6348	0.375 (0.008–18.268)	0.6205
18.50–24.99	1.690 (1.009–2.831)	**0**.**0460**	1.424 (0.699–2.901)	0.3303	0.732 (0.379–1.414)	0.3524	1.060 (0.498–2.256)	0.8797
25.00–29.99	1.727 (1.072–2.783)	**0**.**0247**	2.688 (1.600–4.517)	<**0**.**0001**	0.815 (0.483–1.373)	0.4414	0.698 (0.371–1.313）	0.2652
≥30.00	1.296 (0.901–1.864)	0.1617	1.277 (0.867–1.881)	0.2166	0.784 (0.526–1.169)	0.2330	1.827 (1.228–2.719)	**0**.**0029**
PIR								
<1.00	3.477 (2.209–5.472)	<**0**.**0001**	2.294 (1.251–4.205)	**0**.**0073**	0.539 (0.2665–1.097)	0.0882	1.955 (1.032–3.704)	**0**.**0398**
1.00–1.99	0.892 (0.538–1.480)	0.6588	1.403 (0.849–2.320)	0.1867	0.824 (0.485–1.399)	0.4737	1.154 (0.663–2.008)	0.6125
≥2.00	1.108 (0.739–1.660)	0.6199	1.419 (0.922–2.185)	0.1118	0.916 (0.629–1.333)	0.6453	1.135 (0.729–1.768)	0.5740
Education level								
High school or below	1.603 (1.1552–2.230)	**0**.**0051**	1.528 (1.031–2.267)	**0**.**0349**	0.648 (0.425–0.986)	**0**.**0427**	1.433 (0.930–2.207)	0.1029
Some college	1.191 (0.754–1.883)	0.4534	1.755 (1.101–2.798)	**0**.**0181**	1.1667 (0.744–1.831)	0.5008	1.372 (0.851–2.210)	0.1944
College graduate or above	1.521 (0.746–3.100)	0.2488	1.436 (0.620–3.329)	0.3987	0.690 (0.339–1.403)	0.3053	0.884 (0.377–2.072)	0.7767

Bold values indicate statistical significance.

OR, odd ratio; 95% CI, 95% confidence interval; Ref, reference; BMI, body mass index; PIR, the ratio of family income to poverty.

All data were adjusted for smoking exposure, drinking exposure, hypertension history, diabetes history, COPD history, and CKD history.

**Figure 4:**
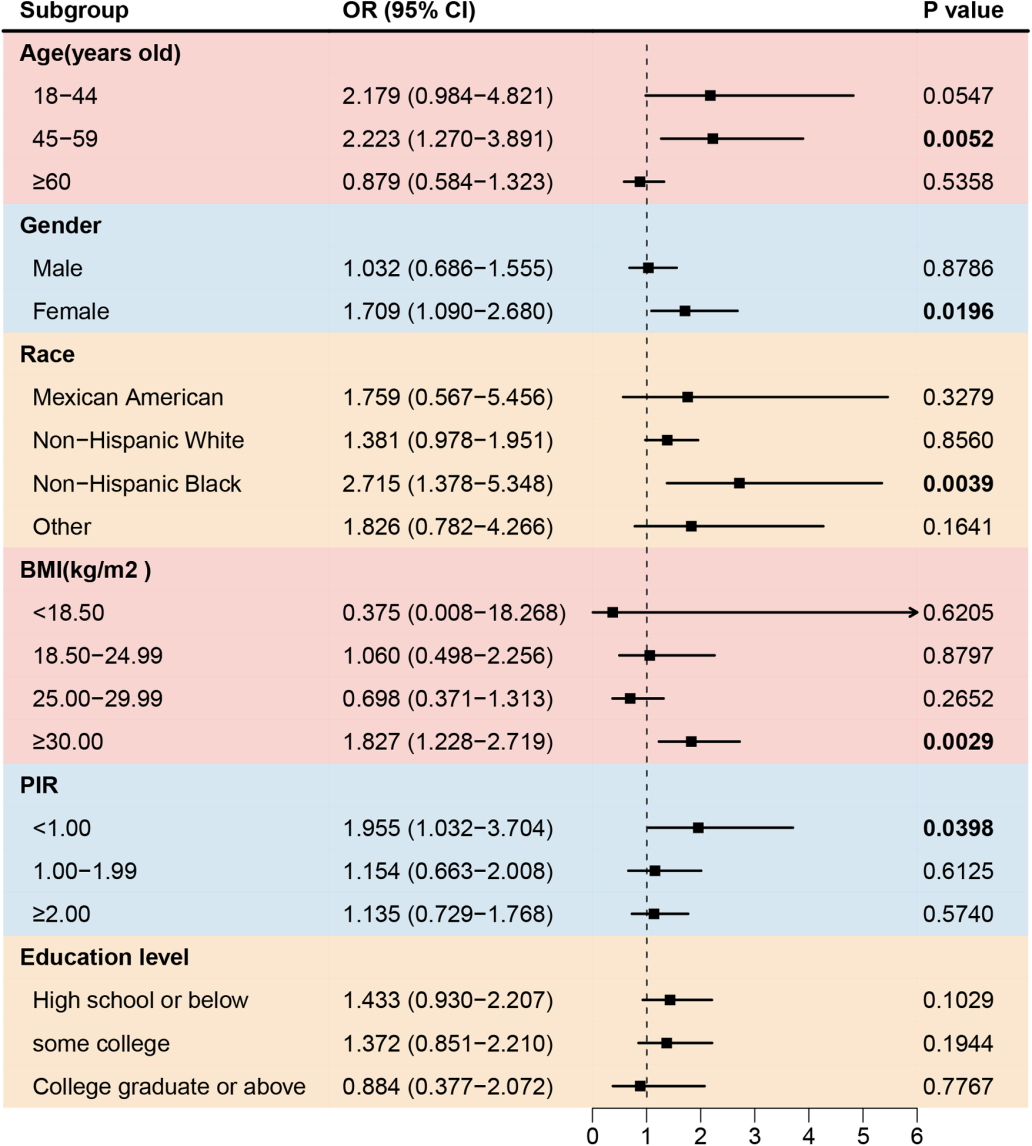
Forest plot of association between asthma and the prevalence of angina. BMI, body mass index; PIR, the ratio of family income to poverty.

**Figure 5:**
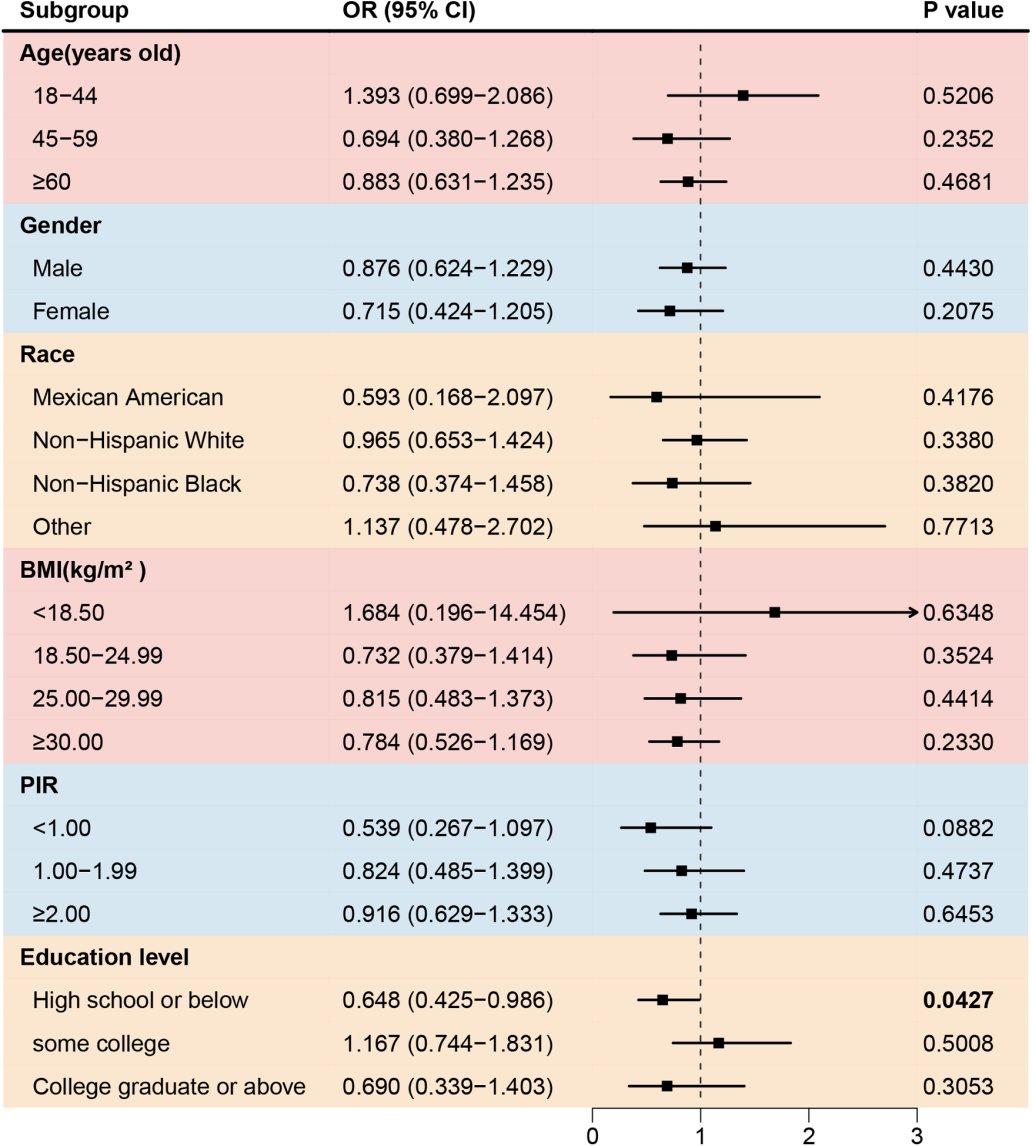
Forest plot of association between asthma and the prevalence of coronary artery disease. BMI, body mass index; PIR, the ratio of family income to poverty.

Asthma also increased the prevalence of angina among females, non-Hispanic Blacks, participants aged 45–59 years old, with a BMI ≥ 30.00 kg/m^2^, and with a PIR < 1.00 (Figure 4). However, the prevalence of coronary artery disease was negative associated with asthma in participants with low education level (Figure 5).

## 4 Discussion

The findings of this study indicate a strong association between asthma and CVD, especially stroke, congestive heart failure. The subgroup analyses further validated the associations between asthma and CVD in several age groups, ethnicities, and PIR levels and in both genderes. These findings provide evidence for implementing national public health initiatives and control measures. This study also enhances public awareness of the interplay between respiratory diseases and CVD.

Stroke, congestive heart failure, coronary heart disease, and angina are among the most common conditions affecting the elderly population. These diseases share risk factors with bronchial asthma including advanced age, obesity, depression, and smoking history (20). Asthma may contribute to the development of stroke by influencing the coagulation processes. Acute asthma exacerbations can lead to hypoxemia, which triggers the production of thrombogenic factors and impairs endothelial function (21). Bazan-Socha et al. (22) reported that individuals with asthma exhibited significantly increased thrombin generation and impaired fibrinolysis. Inflammation is another key factor linking asthma and stroke. A recent study (23) confirmed a significant association between increased levels of the Systemic Immune-Inflammation Index and Systemic Inflammation Response Index and an increased prevalence of stroke in patients with asthma. Interleukin-33 (IL-33), which is elevated in patients with asthma, promotes Th2-type immune responses (24, 25). Interestingly, the IL-13 levels are significantly elevated in patients with acute ischemic stroke and are positively correlated with infarct size (26). In this study, a significant increase in stroke prevalence was observed among participants with asthma aged 18–44 years old, participants with a BMI 18.50–29.99 kg/m^2^, participants with a low education level, participants with a PIR < 1.00.

Previous studies (11, 27) have reported that patients with asthma have a 2.14-fold increased risk of heart failure [95% CI (2.06–2.22)] and a 1.44-fold increased risk of angina [95% CI (1.17–1.77)]. Asthma may contribute to the development of heart failure by elevating the IgE levels. Cardiac mast cells respond to IgE-mediated inflammatory stimuli by releasing large amounts of inflammatory mediators that can damage the normal structure and function of the heart (28). Moreover, asthma medications, including corticosteroids and beta-adrenergic agonists, have adverse effects on the cardiovascular system (29). A meta-analysis (30) found that beta-adrenergic agonists significantly increase the risk of CVD [RR: 2.54; 95% CI (1.59–4.05)]. In this study, positive associations between asthma and congestive heart failure were observed in individuals aged ≥45 years old, especially among non-Hispanic-white or black groups, individuals with a BMI 25.00–29.99 kg/m^2^, and participants with a PIR < 1.00. Asthma also significantly increases the prevalence of angina heart failure in female, non-Hispanic black, and participants with an age 45–59 years old, a BMI ≥ 30.00 kg/m^2^, participants with a PIR < 1.00.

The association between asthma and coronary heart disease (CHD) remains controversial. A meta-analysis (31) demonstrated that asthma significantly increases the risk of myocardial infarction (RR 1.39; 95% CI: 1.16–1.66, *I*^2^ = 59.3%; *p* < 0.001), a finding consistent with several large population-based studies from the Denmark (32), and Chinese Taiwan region (33). However, one observational study and a Mendelian randomization analysis (13) denied an association between asthma and CHD. The probability of developing CHD varies among different subgroups of asthma patients. Study form Taiwan (33) found that males had a higher risk of cardiovascular mortality. Research from the United States (11) identified allergies as a risk factor for CHD, but not allergic asthma. The Copenhagen study (32) observed an increased risk of asthma-related CHD only among smokers. In our study, we found that the risk of CHD was decreased only among asthma patients with a lower education level.

Compared with other studies, this research is a cross-sectional study based on the U.S. population, with a large sample size and high data reliability from the NHANES database. We used an in-depth analytical approach to adjust for a range of potential confounding factors, including sociodemographic factors, lifestyle behaviors, and comorbidities, which strengthened our ability to assess the robustness of the asthma-cardiovascular disease (CVD) relationship in the U.S. population. This study also investigated the association between asthma and CVD in different subgroups, which may help raise public awareness of cardiovascular risks among the asthma population in the U.S. However, there are some limitations. As a cross-sectional study, it cannot infer causal relationships between asthma and CVD. Additionally, the study has geographical limitations, and further research across multiple regions would be meaningful. The mechanisms through which asthma affects CVD still require further exploration.

## 5 Conclusion

Asthma significantly increases the prevalence of stroke, congestive heart failure. Patients with asthma should be monitored for CVD, including stroke and congestive heart failure.

## Data Availability

Publicly available datasets were analyzed in this study. This data can be found here: https://wwwn.cdc.gov/nchs/nhanes/Default.aspx.
